# Dynamics of Fractional Delayed Reaction-Diffusion Equations

**DOI:** 10.3390/e25060950

**Published:** 2023-06-16

**Authors:** Linfang Liu, Juan J. Nieto

**Affiliations:** 1 Department of Mathematics, Northwest University, Xi’an 710127, China; liulinfang2020@nwu.edu.cn; 2 Departamento de Análise Matemática, Estatística e Optimización, Universidade de Santiago de Compostela, 15782 Santiago de Compostela, Spain

**Keywords:** fractional reaction–diffusion equations, bounded variable delay, generalized fractional derivative, generalized comparison principal, global attracting sets

## Abstract

The long-term behavior of the weak solution of a fractional delayed reaction–diffusion equation with a generalized Caputo derivative is investigated. By using the classic Galerkin approximation method and comparison principal, the existence and uniqueness of the solution is proved in the sense of weak solution. In addition, the global attracting set of the considered system is obtained, with the help of the Sobolev embedding theorem and Halanay inequality.

## 1. Introduction

In the present paper, we focus on the asymptotical behavior of the following fractional reaction–diffusion equation with variable delays in a bounded domain Ω:

(1) $$D_{t}^{\alpha }u-\nu \Delta u=f\left(u\right)+g\left(t,u_{t}\right),\;\mathrm{in}\;\left(0,T\right)\times \Omega ;\;$$

(2) u=0,on(0,T)×∂Ω;

(3) u(θ,x)=ϕ(θ,x),θ∈[−h,0],x∈Ω,

where $D_{t}^{\alpha }$ is the generalized Caputo derivative (see [1] for generalized fractional derivative, and [2,3] for the classical fractional derivative) with order α∈(0,1), $u_{t}\left(\theta \right):=u\left(t+\theta \right),\theta \in \left[-h,0\right]$, h>0 is a constant, $\Omega \subset R^{n}$ is a bounded open set with smooth boundary condition ∂Ω, ν is a positive constant, *u* is the unknown function, function ϕ defined on [−h,0] is the initial value, the external forcing term *f* is non-delayed, and the external forcing term *g* possesses some hereditary property.

Hereditary characteristics appear in many disciplines, such as chemistry, economics, biosciences, and physiology; they also relate to many realistic problems: for instance, the feedback control problem; matching market with imperfect information; immune systems; and soft matter with viscoelasticity [4,5]. This property, which sometimes is called after-effect, can appear as a variable delay or as a distributed delay, including bounded and unbounded delay, etc. On the other hand, using fractional calculus to model the hereditary effect is another common skill, which has been extensively applied in many sciences [2,3,6]. It is well-known that the early definition of fractional order calculus was introduced by L’Hospital in the late 17th century, and has become famous in practical applications only within the last few decades. As shown in [2], there are several ways to define a fractional derivative, and probably the most frequently used are the so-called Riemann–Liouville derivative and the Caputo derivative [3]. The Caputo derivative was defined in [7], to describe the memory effect for unelastic materials.

The authors of [1] raised a generalized definition of Caputo derivatives from t=0 of order α∈(0,1), with the help of a convolution group, and built a convenient framework for investigating time-fractional differential equations with boundary and initial values.

There are many results for non-local reaction–diffusion equations, which are often used to model anomalous diffusion (such as sub-diffusion or super-diffusion) in fractal media: for example, [5,8]. In [9,10], time-fractional advection–reaction–diffusion equations were studied; Ref. [11] discussed the analytical solution for time–space fractional reaction–diffusion equations; Ref. [12] applied a fractional reaction–diffusion system to image denoising; while [13,14] investigated the numerical methods for equations with fractional derivatives. Furthermore, Refs. [15,16,17,18] studied predator–prey-like problems, by applying a spatial Caputo operator, and, in [19], the authors studied the numerical simulation of chaotic evolution with a time-fractional generalized Caputo derivative. Nevertheless, most of the available works, including those mentioned above, mainly focus on non-delay cases, and concern the well-posedness of a solution/mild solution or the regularity. There are few works on the asymptotical behavior of solutions, and even fewer works about time-fractional reaction–diffusion equations with delay, nor about the existence of, or the long-term behavior of, weak solutions. Generally, for fractional partial differential equations, this discussion is still limited, due to the lack of effective tools, even though [20,21] have studied fractional partial differential equations in some special cases.

A common technique for investigating the long-term quality of weak solutions of traditional nonlinear partial differential equations is to obtain some estimates of the energy equation, and then to employ classical compactness theorems, i.e., the Arzelà–Ascoli theorem, the Aubin–Lions lemma, etc. Unfortunately, this method appears to be invalid, because of the appearance of a delay term, and the lack of a fundamental compactness theorem for the corresponding fractional case. However, by using generalized Halanay inequalities, the authors of [22] discussed the dissipativity of Volterra functional differential equations, while the authors of [23] generalized the Henry–Gronwall-type integral inequalities with delay, and applied them to fractional delay differential equations.

Motivated by [22,23], we investigated the dynamics of fractional Navier–Stokes equations without delay in our former work [24], in which we obtained the existence and uniqueness of weak solutions of the system, but could not establish the existence of a global attracting set: indeed, it was complicated even to prove the existence of a weak solution in delay cases, let alone the existence of a global attracting set. To obtain the existence of a global attracting set for a fractional dynamics system, we study, in the present work, the limited behavior of generalized Caputo reaction–diffusion equations with variable delays. The structure of this article is arranged as follows. The next section will recollect some definitions about fractional calculus and lemmas, which will be needed later in the study. Section 3 focuses on the well-posedness of (1)–(3): namely, the existence and uniqueness of solutions in the weak sense, by Galerkin approximation. The existence of a global attracting set is shown in Section 4.

## 2. Preliminaries

In this section, we recall some concepts of generalized fractional calculus for functions valued in normed vector spaces, as introduced in [1,25]. Then, for the sake of the completeness of the work, and to make the paper easier to read, we recollect some notations and abstract spaces. In addition, an example of delay, some lemmas, and propositions that will be used in our later discussion are also provided in this section. We begin with the definition of a fractional integral; readers are referred to [1,2,3] for more details. Note that *c* and *C* are positive constants, which may vary in different lines.

**Definition** **1** ([3])**.** *The Riemann–Liouville fractional integral of order α∈(0,1) for a locally integrable function $u:R^{+}\to R$ is defined as*

$$\begin{array}{c} \left[I_{\alpha }u\right]\left(t\right)=\frac{1}{\Gamma (\alpha )}\int_{0}^{t}{\left(t-s\right)}^{\alpha -1}u\left(s\right)ds,\;\;t>0, \end{array}$$

*where $\Gamma \left(\alpha \right)=\int_{0}^{\infty }x^{\alpha -1}e^{-x}dx$.*

**Definition** **2** ([1])**.** *Suppose X is a Banach space, and $u\in L_{loc}^{1}\left(\left(0,T\right);X\right)$ is a locally integrable function; if there is $u_{0}\in X$ satisfying*

$$\begin{array}{c} \lim_{t\to 0_{+}}\frac{1}{t}\int_{0}^{t}{∥u\left(s\right)-u_{0}∥}_{X}ds=0, \end{array}$$

*then we say $u_{0}$ is the right limit of function u at t=0, denoted as $u\left(0_{+}\right)=u_{0}$. By using the same approach, we define $u(T^{-})$ to be the left limit of u at t=T, i.e., $u(T^{-})\in X$ satisfying*

$$\begin{array}{c} \lim_{t\to T^{-}}\frac{1}{T-t}\int_{0}^{t}{∥u\left(s\right)-u_{T}∥}_{X}ds=0. \end{array}$$

In fact, the fractional integral defined in Definition 1 can be characterized by the convolution between $g_{\alpha }\left(t\right)=\frac{H\left(t\right)t^{\alpha -1}}{\Gamma (\alpha )}$, which is called the kernel, and H(t)u(t) on R, in which

$$H\left(t\right)=\left\{\begin{array}{c} 1,\;\;t\geq 0,\\ 0,\;\;t<0 \end{array}\right.$$

is the standard Heaviside step function (see [26] for more information). It is not hard to check that the integral operators $I_{\alpha }$ satisfy the semigroup property; moreover, each $I_{\alpha }$ is also a continuous linear transformation from Banach space $L^{1}\left(0,T\right)$ to $L^{1}\left(0,T\right)$. Motivated by these two facts about $I_{\alpha }$, Li and Liu [1] proposed a generalized Caputo derivative, which is consistent with various definitions in the mentioned literature, while revealing the underlying group structure. This group property makes many properties of this generalized Caputo derivative natural.

Before introducing this generalized Caputo derivative, we need to use the below distributions $\left\{g_{\alpha }\right\}$ as the convolution kernels for α∈(−1,1):

$$g_{\alpha }\left(t\right):=\left\{\begin{array}{c} \;\frac{H\left(t\right)t^{\alpha -1}}{\Gamma (\alpha )},\;\;\alpha >0,\\ \;\delta (t),\;\;\;\;\;\alpha =0,\\ \;\frac{D(H\left(t\right)t^{\alpha })}{\Gamma (1+\alpha )},\;\;\alpha \in \left(-1,0\right), \end{array}\right.$$

where δ is the usual Dirac distribution, and *D* means the distributional derivatives. Then, the fractional integral operator $I_{\alpha }$ can be expressed as

$$\begin{array}{c} \left[I_{\alpha }u\right]\left(t\right):=g_{\alpha }*\left(H\left(t\right)u\left(t\right)\right). \end{array}$$

Given $f,g\in L_{loc}^{1}\left(0,T\right)$, the convolution between *f* and *g* as

$$f\left(t\right)*g\left(t\right)=\int_{0}^{t}f\left(s\right)g\left(t-s\right)ds,\;\;t\in \left(0,T\right).$$

We denote by (·,·) and |·| the scalar product and norm, respectively, in $L^{2}\left(\Omega \right)$. The norm in $L^{p}\left(\Omega \right)$ is written as ${∥\cdot ∥}_{L^{p}\left(\Omega \right)}$; we also use (·,·) to denote the duality between $L^{p}\left(\Omega \right)$ and $L^{q}\left(\Omega \right)$, where $\frac{1}{p}+\frac{1}{q}=1$, with 1≤p,q<∞. In $H_{0}^{1}\left(\Omega \right)$, we use as (equivalent) scalar product ((·,·))=(∇·,∇·), with the corresponding norm ∥·∥, and the duality between $H_{0}^{1}\left(\Omega \right)$ and its dual $H^{-1}\left(\Omega \right)$ is written as 〈·,·〉. We use ${∥\cdot ∥}_{*}$ for the norm in $H^{-1}\left(\Omega \right)$.

In addition, we denote $u_{t}$ as the element of Banach space $C_{H}:=C\left(\left[-h,0\right];L^{2}\left(\Omega \right)\right)$ defined by $u_{t}\left(\theta \right):=u\left(t+\theta \right)$, θ∈[−h,0]. The norm of Banach space $C_{H}$ is defined as $∥u_{t}{∥}_{C_{H}}=\underset{-h\leq \theta \leq 0}{\sup}\left|u\left(t+\theta \right)\right|$.

To this end, the generalized Caputo derivative is given as

**Definition** **3** ([1])**.** *Let α∈(0,1). Consider that $u\in L_{loc}^{1}\left(0,T\right)$ possesses a right limit $u\left(0_{+}\right)=u_{0}$ at t=0, in the sense of Definition 2. The left Caputo derivative of u with order α is a distribution in $D^{\prime }\left(-\infty ,T\right)$ with support in [0,T), given by*

$$\begin{array}{c} D_{t}^{\alpha }u:=I_{-\alpha }u-u_{0}g_{1-\alpha }=g_{-\alpha }*\left[\left(\left(u-u_{0}\right)H\left(t\right)\right)\right]. \end{array}$$

**Definition** **4** ([25])**.** *Let α∈(0,1). Consider that $u\in L_{loc}^{1}\left(-\infty ,T\right)$ possesses a left limit $u\left(T^{-}\right)=u_{T}$ at t=T, in the sense of Definition 2. The right Caputo derivative of u with order α is a distribution in $D^{\prime }\left(R\right)$ with support in (−∞,T], given by*

$$\begin{array}{c} {\tilde{D}}_{c;T}^{\alpha }u:={\tilde{g}}_{-\alpha }*\left[H\left(T-t\right)\left(u-u_{T}\right)\right]. \end{array}$$

In this work, we investigate problems (1)–(3) in weak topology instead of norm topology: for this, the definition of the weak generalized Caputo derivative is also needed, which is defined by Definition 2.4 in [25].

**Definition** **5** ([25])**.** *Suppose X is a Banach space, and $u\in L_{loc}^{1}\left(\left[0,T\right);X\right)$. Let $u_{0}\in X$. We define the weak Caputo derivative of u associated with initial condition $u_{0}$ to be $D_{t}^{\alpha }u\in D^{\prime }\left(R\right)$, such that for any test function $v\in C_{c}^{\infty }\left(\left(-\infty ,T\right);R\right)$,*

$$\begin{array}{c} \langle v,D_{t}^{\alpha }u\rangle :=\int_{-\infty }^{T}\left({\tilde{D}}_{c;T}^{\alpha }v\right)\left(u-u_{0}\right)H\left(t\right)dt=\int_{0}^{T}\left({\tilde{D}}_{c;T}^{\alpha }v\right)\left(u-u_{0}\right)dt. \end{array}$$

We also need to make the following assumptions, based on the external term $f\left(u\right)\in C^{1}\left(R\right)$ and the delay term $g(t,u_{t})$. There exist positive constants $\alpha_{i}$, i=1,2,3,4, $\beta_{1}$, $\beta_{2}$ and p>2, such that

(4) $$-\beta_{1}-\alpha_{1}{\left|s\right|}^{p}\leq f\left(s\right)s\leq \beta_{2}-\alpha_{2}{\left|s\right|}^{p},\;\;\forall s\in R;$$

(5) $$f^{\prime }\left(s\right)\leq \alpha_{3},\;\;\forall s\in R;$$

(6) $$\left|f\left(s\right)\right|\leq \alpha_{4}{\left(1+|s\right|}^{p-1}),\;\;\forall s\in R.$$

For the assumptions based on the delay terms *g* with $g:\left[0,T\right]\times C_{H}\to R$, we may assume:

(g1) $\forall \;\xi \in C_{H}$, the mapping $\left[0,T\right]∋t\mapsto g\left(t,\xi \right)\in {\left(L^{2}\left(\Omega \right)\right)}^{2}$ is measurable;
(g2) g(·,0)=0;
(g3) There exists a positive constant $L_{g}$, such that ∀t∈[τ,T], $\forall \;\xi ,\eta \in C_{H}$,
  $$\begin{array}{c} \left|g\left(t,\xi \right)-g\left(t,\eta \right)\right|\leq L_{g}{∥\xi -\eta ∥}_{C_{H}}. \end{array}$$

**Example** **1** **(A forcing term with bounded variable delay).** *Let $F:\left[t_{0},T\right]\times R^{2}\to R^{2}$ be a measurable function, such that F(t,0)=0, $\forall t\in [t_{0},T]$, and suppose that there exists a positive constant M satisfying*

$$\begin{array}{c} {\left|F\left(t,u\right)-F\left(t,v\right)\right|}_{R^{2}}\leq M{\left|u-v\right|}_{R^{2}},\;\mathrm{for}\;\mathrm{any}\;u,v\in R^{2}. \end{array}$$

*Consider a measurable function ρ(·):[0,+∞)→[0,h], and define g(t,ξ)(x)=F(t,ξ(−ρ(t))(x)) for each $\xi \in C_{H}$, x∈Ω and $t\in [t_{0},T]$; then, the delayed term g becomes*

$$\begin{array}{c} g(t,\xi )=F(t,\xi (-\rho (t))). \end{array}$$

*Henceforth, we take $g\left(t,u_{t}\right)=g\left(u\left(t-\tau \left(t\right)\right)\right)$, where the delay function τ(t):R→[0,h] is continuous, and take A=−Δ. An equivalent abstract formulation to problems (1)–(3) is*

(7) $$\begin{array}{c} D_{t}^{\alpha }u+\nu Au=f\left(u\right)+g\left(t,u_{t}\right),\;\;\forall t\geq 0; \end{array}$$

(8) $$\begin{array}{c} u(t)=\phi (t),\;t\in [-h,0]. \end{array}$$

*As noted above, we study problems (7) and (8) in weak topology, i.e., the asymptotic behavior of a weak solution will be investigated. The definition of a weak solution to problem (7) and (8) is defined as*

**Definition** **6** ([25])**.** *Given an initial value $\phi \in C(\left[-h,0\right];L^{2}\left(\Omega \right))$, a function $u\in C\left(\left[-h,0\right];L^{2}\left(\Omega \right)\right)\cap L^{p}\left(0,T;L^{p}\left(\Omega \right)\right)\cap L^{2}\left(0,T;H_{0}^{1}\left(\Omega \right)\right)$ with $u_{0}=\phi$ is called a weak solution to problem (7) and (8) in the interval [−h,T], if for any $v\in H_{0}^{1}\left(\Omega \right)\cap L^{p}\left(\Omega \right)$,*

$$\left(D_{t}^{\alpha }u\left(t\right),v\right)+\nu \left(\left(u\left(t\right),v\right)\right)=\left(f\left(u\right),v\right)+\left(g\left(t,u_{t}\right),v\right),$$

*where the equation must be understood in the sense of $D^{\prime }\left(R\right)$.*

The following auxiliary lemmas will be needed in this work.

**Lemma** **1** (See [25,27])**.** *For any absolutely continuous function u(t) defined on [0,T], it holds that*

$$\begin{array}{c} u\left(t\right)D_{t}^{\alpha }u\left(t\right)\geq \frac{1}{2}D_{t}^{\alpha }u^{2}\left(t\right),\;\alpha \in \left(0,1\right). \end{array}$$

**Proposition** **1** (The generalized comparison principle Proposition 3 in [24]). *Assume that for any absolutely continuous function u(t) defined on [0,T], it holds that*

$$\begin{array}{cc} \; & D_{t}^{\alpha }u\left(t\right)\leq -au\left(t\right)+bu\left(t-\tau \left(t\right)\right)+c,\;\;0<t<T,\\ \; & u(t)=\varphi (t),\;\;-h\leq t\leq 0, \end{array}$$

*and for absolutely continuous w(t) defined on [0,T], the following fractional dynamical system*

$$\begin{array}{cc} \; & D_{t}^{\alpha }w\left(t\right)=-aw\left(t\right)+bw\left(t-\tau \left(t\right)\right)+c,\;\;0<t<T,\\ \; & w(t)=\varphi (t),\;\;-h\leq t\leq 0, \end{array}$$

*where a,b,c are positive constants. Then, for all t≥−h, we have*

$$\begin{array}{c} u(t)\leq w(t). \end{array}$$

**Proposition** **2** (Theorem 4.2 in [25]). *Let T>0, α∈(0,1) and p≥1. Let M,X,Y be Banach spaces. M↪X compactly and X↪Y continuously. Assume $W\subset L_{loc}^{1}\left(\left(0,T\right);M\right)$ satisfies:*

*(i)*  *There exists $r_{1}\in \left[1,\infty \right)$ and C>0, such that for any u∈W,*
  $$\begin{array}{c} \underset{t\in (0,T)}{\sup}I_{\alpha }{(∥u∥}_{M}^{r_{1}})=\underset{t\in (0,T)}{\sup}\frac{1}{\Gamma (\alpha )}\int_{0}^{t}{\left(t-s\right)}^{\alpha -1}{∥u∥}_{M}^{r_{1}}\left(s\right)ds\leq C; \end{array}$$
*(ii)*  *There exists $p_{1}\in \left(p,\infty \right]$, and W is bounded in $L^{p_{1}}\left(\left(0,T\right);X\right)$;*
*(iii)*  *There exists $r_{2}\;\in \;\left[1,\infty \right)$ and C>0, such that for any u∈W, with right limit $u_{0}$ at t=0, it holds that*
  $$\begin{array}{c} ∥D_{t}^{\alpha }{u∥}_{L^{r_{2}}\left(\left(0,T\right);Y\right)}\leq C. \end{array}$$

*Then, W is relatively compact in $L^{p}\left(\left(0,T\right);X\right).$*

**Proposition** **3** (An improvement in Proposition 3.5 in [25]). *Assume that Y is a reflexive space, α∈(0,1) and T>0. Suppose $u^{n}\to u$ in $L^{p}\left(\left(0,T\right);Y\right)$, p≥1. If there is an assignment of initial values $u_{0,n}$ for $u^{n}$, such that the weak Caputo derivatives $D_{t}^{\alpha }u^{n}$ are bounded in $L^{r}\left(\left(0,T\right);Y\right)$(r∈[1,∞)), then*

*(i)*  *There is a sub-sequence, such that $u_{0,n}$ converges weakly to some value $u_{0}\in Y;$*
*(ii)*  *If r>1, there exists a sub-sequence, such that $D_{t}^{\alpha }u^{nk}$ converges to v, and $u_{0,n_{k}}$ converges to $u_{0}$, in the sense of weak topology, and v is the Caputo derivative of u with initial value $u_{0}$, so that*
  $$\begin{array}{c} u=u_{0}+\frac{1}{\Gamma (\alpha )}\int_{0}^{t}{\left(t-s\right)}^{\alpha -1}v\left(s\right)ds. \end{array}$$

*Furthermore, if r≥1, then $u\left(0_{+}\right)=u_{0}$ in Y.*

**Proof.**  Readers are referred to [24] for the proof. □

**Lemma** **2** (A generalized Halanay inequality, Lemma 4 in [8])**.** *supposes that the continuous function v(t) is non-negative, and satisfies*

$$\begin{array}{c} D_{t}^{\alpha }v\left(t\right)\leq \gamma +av\left(t\right)+b\underset{t-\tau (t)\leq s\leq t}{\sup}v\left(s\right),\;\;0<t<T, \end{array}$$

*and*

$$\begin{array}{c} v(t)=|\varphi (t)|,\;\;-\sigma \leq t\leq 0, \end{array}$$

*where γ>0 is a constant, and a+b≠0, $\sigma =-\underset{t\geq 0}{\inf}\left(t-\tau \left(t\right)\right)>0$, and where the delay function τ(t)∈[0,h]. If a+b<0, then the following estimate,*

(9) $$v\left(t\right)\leq -\frac{\gamma }{a+b}+ME_{\alpha }\left(\lambda^{*}t^{\alpha }\right),$$

*holds for all t≥τ(t), where $M=\underset{-h\leq t\leq 0}{\sup}{∥\varphi \left(t\right)∥}^{2}$, and the parameter $\lambda^{*}$ is defined by*

$$\begin{array}{c} \lambda^{*}=\underset{t-\tau (t)\geq 0}{\sup}\left\{\lambda :\lambda -a-b\frac{E_{\alpha }\left(\lambda {\left(t-\tau \left(t\right)\right)}^{\alpha }\right)}{E_{\alpha }\left(\lambda t^{\alpha }\right)}=0\right\}, \end{array}$$

*and it holds that $\lambda^{*}\in \left[a+b,0\right]$.*

*Furthermore, if the delay is bounded, i.e., $0\leq \tau \left(t\right)\leq \tau_{0}$ for some positive constant $\tau_{0}$, then the parameter $\lambda^{*}$ defined by*

$$\begin{array}{c} \lambda^{*}=\underset{t-\tau (t)\geq 1}{\sup}\left\{\lambda :\lambda -a-b\frac{E_{\alpha }\left(\lambda {\left(t-\tau \left(t\right)\right)}^{\alpha }\right)}{E_{\alpha }\left(\lambda t^{\alpha }\right)}=0\right\}, \end{array}$$

*which is strictly negative, i.e., there exist some positive constants $\epsilon_{0}$ satisfying $a+b<-\epsilon_{0}$, such that $\lambda^{*}\in \left[a+b,-\epsilon_{0}\right]$, and the inequality in (9) holds for all t, such that t≥τ(t)+1.*

**Proof.**  The proof is given in our previous work [24]. □

**Lemma** **3** (Bellman–Gronwall Lemma [28] p. 252)**.** *Let T>0, $g\in L^{1}\left(0,T\right)$ and g≥0 a.e., $C_{1}>0$, $C_{2}>0$ are constants. If $\varphi \in L^{1}\left(0,T\right)$, φ≥0 a.e., with $g\varphi \in L^{1}\left(0,T\right)$ and*

$$\begin{array}{c} \varphi \left(t\right)\leq C_{1}+C_{2}\int_{0}^{t}g\left(s\right)\varphi \left(s\right)ds,\;\;a.e.\;t\in \left(0,T\right), \end{array}$$

*then,*

$$\begin{array}{c} \varphi \left(t\right)\leq C_{1}\exp\left\{C_{2}\int_{0}^{t}g\left(s\right)ds\right\},\;\;a.e.\;t\in \left(0,T\right). \end{array}$$

## 3. Well-Posedness

In this section, we show the existence and uniqueness of weak solutions to problems (7) and (8) by Galerkin approximations. Denote

$$\lambda_{1}=\underset{v\in V\setminus \{0\}}{\inf}\frac{{∥v∥}^{2}}{{\left|v\right|}^{2}}>0.$$

For the existence of a weak solution, we have the following result:

**Theorem** **1.** *Suppose that (4)–(6) and (g1)–(g3) hold true, then for any $\phi \in C_{H}$, system (7) and (8) has a unique weak solution.*

**Proof.**  The proof is split into five steps.

Step 1. The Galerkin approximation. From the classical spectral theory of elliptic operators, we know that the Laplacian operator A=−Δ has a sequence of eigenvalues ${\left\{\lambda_{j}\right\}}_{j\geq 1}$ with corresponding eigenfunctions ${\left\{w_{j}\right\}}_{j\geq 1}\subset V$, which form a Hilbert basis of *H*, and are dense on *V*. Consider the linear subspace $V_{m}=\mathrm{span}\left\{w_{1},w_{2},\cdots ,w_{m}\right\}$ and the projector $P_{m}:H\to V_{m}$ given by $P_{m}u=\sum_{j=1}^{m}\left(u,w_{j}\right)w_{j}$, and define $u^{\left(m\right)}\left(t\right)=\sum_{j=1}^{m}\gamma_{m,j}\left(t\right)w_{j}$, where the superscript *m* will be used instead of (m), for short, as no confusion is possible with powers of *u*, and where the coefficients $\gamma_{m,j}\left(t\right)$ are required, to satisfy the initial value problem:

(10) $$\begin{array}{c} \left(D_{t}^{\alpha }u^{m}\left(t\right),w_{j}\right)+\nu \left(\left(u^{m}\left(t\right),w_{j}\right)\right)=\left(f\left(u\right),w_{j}\right)+\left(g\left(t,u_{t}^{m}\right),w_{j}\right),\;\;1\leq j\leq m,\\ u^{m}\left(\theta \right)=P_{m}\phi \left(\theta \right),\;\theta \in \left[-h,0\right]. \end{array}$$

It is obvious that the above system is fractional order differential equations with bounded delay, which fulfills the conditions for the existence and uniqueness of a local solution (readers are referred to Theorem 3.1 in [29] for details); therefore, we obtain (10) having a unique local solution defined in $\left[0,t_{m}\right)$, with $0\leq t_{m}\leq T$. Next, we find a priori estimates, and ensure that the solutions $u^{m}$ exist on [0,T]. We assume that $M=\underset{-h\leq t\leq 0}{\sup}{\left|\phi \left(t\right)\right|}^{2}.$

Step 2. A priori estimates. Taking the inner product of (10) with $\gamma_{m,j}\left(t\right)$, j=1,…,m, summing up, and applying Lemma 1, Cauchy–Schwartz inequality and Young’s inequality, we obtain

$$\begin{array}{cc} \frac{1}{2}D_{t}^{\alpha }|u^{m}{\left(t\right)|}^{2}+\nu ∥u^{m}{\left(t\right)∥}^{2}+\alpha_{2}{∥u∥}_{p}^{p} & \leq \beta_{2}|\Omega |+|g\left(t,u_{t}^{m}\right)\left|\right|u^{m}\left(t\right)|\\ \; & \leq \beta_{2}\left|\Omega \right|+L_{g}{∥u_{t}^{m}∥}_{C_{H}}^{2}. \end{array}$$

Hence,

(11) $$\begin{array}{c} D_{t}^{\alpha }|u^{m}{\left(t\right)|}^{2}+\lambda_{1}\nu |u^{m}{\left(t\right)|}^{2}+\nu ∥\nabla u^{m}{\left(t\right)∥}^{2}+2\alpha_{2}∥u^{m}{∥}_{p}^{p}\leq 2\beta_{2}\left|\Omega \right|+2L_{g}{∥u_{t}^{m}∥}_{C_{H}}^{2}. \end{array}$$

Multiplying (11) by $I_{\alpha }$, and allowing p=1+α, $q=1+\frac{1}{\alpha }$, we find

$$\begin{array}{cc} \; & |u^{m}{\left(t\right)|}^{2}+\frac{\nu }{\Gamma (\alpha )}\int_{0}^{t}{\left(t-s\right)}^{\alpha -1}(\lambda_{1}|u^{m}{\left(s\right)|}^{2}+∥\nabla u^{m}{\left(s\right)∥}^{2})ds+\frac{2\alpha_{2}}{\Gamma (\alpha )}\int_{0}^{t}{\left(t-s\right)}^{\alpha -1}{∥u^{m}\left(s\right)∥}_{p}^{p}ds\\ \; & \leq |u^{m}{\left(0\right)|}^{2}+\frac{2\beta_{2}\left|\Omega \right|}{\Gamma (\alpha +1)}t^{\alpha }+\frac{2L_{g}}{\Gamma (\alpha )}\int_{0}^{t}{\left(t-s\right)}^{\alpha -1}{∥u_{s}^{m}∥}_{C_{H}}^{2}ds\\ \; & \leq |u^{m}{\left(0\right)|}^{2}+\frac{2\beta_{2}\left|\Omega \right|}{\Gamma (\alpha +1)}t^{\alpha }+\frac{2L_{g}}{\Gamma (\alpha )}{\left(\int_{0}^{t}{\left(t-s\right)}^{p(\alpha -1)}e^{ps}ds\right)}^{\frac{1}{p}}{\left(\int_{0}^{t}e^{-qs}{∥u_{s}^{m}∥}_{C_{H}}^{2q}ds\right)}^{1/q}\\ \; & \leq |u^{m}{\left(0\right)|}^{2}+\frac{2\beta_{2}\left|\Omega \right|}{\Gamma (\alpha +1)}t^{\alpha }+\frac{2L_{g}\Gamma \left(\alpha^{2}\right)}{\Gamma (\alpha )}{\left(1+\alpha \right)}^{-\frac{\alpha^{2}}{1+\alpha }}e^{t}{\left(\int_{0}^{t}e^{-qs}{∥u_{s}^{m}∥}_{C_{H}}^{2q}ds\right)}^{1/q}, \end{array}$$

denoted by $A\left(t\right)=|u^{m}{\left(0\right)|}^{2}+\frac{2\beta_{2}\left|\Omega \right|}{\Gamma (\alpha +1)}t^{\alpha }$, $B\left(t\right)=\frac{2L_{g}\Gamma \left(\alpha^{2}\right)}{\Gamma (\alpha )}{\left(1+\alpha \right)}^{-\frac{\alpha^{2}}{1+\alpha }}e^{t}$. Then, we have

$$\begin{array}{c} ∥u_{t}^{m}{∥}_{C_{H}}^{2}\leq A\left(t\right)+B\left(t\right){\left(\int_{0}^{t}{∥u_{s}^{m}∥}^{2q}ds\right)}^{1/q}. \end{array}$$

Therefore,

$$\begin{array}{c} ∥u_{t}^{m}{∥}_{C_{H}}^{2q}\leq 2^{q}A\left(t\right)+2^{q}B^{q}\left(t\right)\left(\int_{0}^{t}{∥u_{s}^{m}∥}_{C_{H}}^{2q}ds\right). \end{array}$$

Using the Gronwall Lemma, we obtain

$$\begin{array}{c} ∥u_{t}^{m}{∥}_{C_{H}}^{2}\leq c\left(A\left(t\right)+B\left(t\right)\int_{0}^{t}A\left(s\right)exp\left(c\int_{s}^{t}B\left(r\right)drds\right)\right),\;\mathrm{for}\;\mathrm{all}\;t\in \left[0,T\right]\;\mathrm{and}\;\theta \in \left[-h,0\right]. \end{array}$$

Thus, we establish that for any T>0, $∥u_{t}^{m}{∥}_{C_{H}}$ is bounded, which implies that the local solution $u^{m}\left(t;\phi \right)$ is in fact a global one. We can also assert that there exists a positive constant *C*, depending on $\nu ,L_{g}$, and *f*, and on *T* and M>0, such that

$$∥u_{t}^{m}{∥}_{C_{H}}^{2}\leq C\left(T,M\right)\;\;\forall t\in \left[0,T\right],\;\;{∥\phi ∥}_{C_{H}}\leq M,\;\;\forall m\geq 1,$$

which also means that $\left\{u^{m}\right\}$ is bounded in $L^{\infty }\left(-h,T;H\right).$

Through the above uniform estimates and (11), we find that

$$\nu \int_{0}^{t}{\left(t-s\right)}^{\alpha -1}∥u^{m}{\left(s\right)∥}^{2}ds\leq {\left|u^{m}\left(0\right)\right|}^{2}+\int_{0}^{t}{\left(t-s\right)}^{\alpha -1}\left(\frac{1}{\nu }{∥f\left(s\right)∥}_{*}^{2}+2L_{g}C\left(T,M\right)\right)ds.$$

We can conclude the existence of another constant (relabelled the same), C(T,M), such that

$$∥u^{m}{∥}_{L^{2}\left(0,T;V\right)}^{2}\leq T^{1-\alpha }\int_{0}^{T}{\left(T-s\right)}^{\alpha -1}{∥u^{m}∥}^{2}ds\leq C\left(T,M\right)\;\;\forall m\geq 1,$$

and

$$∥u^{m}{∥}_{L^{p}\left(0,T;L^{p}\left(\Omega \right)\right)}^{p}\leq T^{1-\alpha }\int_{0}^{T}{\left(T-s\right)}^{\alpha -1}{∥u^{m}∥}_{p}^{p}ds\leq C\left(T,M\right)\;\;\forall m\geq 1,$$

Note that (6), for $\frac{1}{p}+\frac{1}{q}=1$,

$$\begin{array}{cc} ∥f\left(u^{m}\right){∥}_{L^{q}\left(\left(0,T\right);L^{q}\left(\Omega \right)\right)}^{q} & =\int_{0}^{T}\int_{\Omega }{\left|f\left(u^{m}\right)\right|}^{q}dxdt\\ \; & \leq \int_{0}^{T}\int_{\Omega }|\alpha_{4}\left(1+\right|u^{m}{|}^{p-1}{)|}^{q}dxdt\\ \; & \leq c\int_{0}^{T}\int_{\Omega }\left(\right|u^{m}{|}^{q(p-1)}+1)dxdt\\ \; & \leq C(T,M)\;\;\forall m\geq 1. \end{array}$$

On the other hand, $L^{2}\left(0,T;V^{\prime }\right)$ and $L^{q}(0,T;L^{q}\left(\Omega \right)$ are continuously included in $L^{q}\left(0,T;H^{-1}\left(\Omega \right)\right)$. Then, it follows that, from (10),

$$\begin{array}{c} ∥D_{t}^{\alpha }u^{m}{∥}_{*}\leq \nu ∥u^{m}∥+|f\left(u^{m}\right)|+|g\left(t,u_{t}^{m}\right)|, \end{array}$$

which, together with Remark 2.1(ii) and the above estimates imply that $\left\{D_{t}^{\alpha }u^{m}\right\}$ is bounded in $L^{2}\left(0,T;V^{\prime }\right).$ Then, by Proposition 2, we conclude that

$$\begin{array}{c} u^{m}\to u\;\mathrm{strongly}\;\mathrm{in}\;L^{2}\left(\left(0,T\right);L^{2}\left(\Omega \right)\right). \end{array}$$

Step 3. Approximation of initial datum in $C_{H}$. Let us check

(12) $$P_{m}\phi \to \phi \;\;\mathrm{in}\;C_{H}.$$

Assume that $\theta_{m}\to \theta \in \left[-h,0\right]$, then $P_{m}\phi \left(\theta_{m}\right)\to \phi \left(\theta \right)$, since $|P_{m}\phi \left(\theta_{m}\right)-\phi \left(\theta \right)\left|\leq \right|P_{m}\phi \left(\theta_{m}\right)-P_{m}\phi \left(\theta \right)\left|+\right|P_{m}\phi \left(\theta \right)-\phi \left(\theta \right)|\to 0$ as m→∞; therefore, (12) holds true.

Step 4. Compactness results. By Proposition 2, there exists a sub-sequence still relabelled as $\left\{u^{m}\right\}$ that converges to *u* in $L^{2}\left(\left(0,T\right);H\right).$ Using Proposition 3, *u* has a weak Caputo derivative with initial value $u_{0}$, such that

$$\begin{array}{c} D_{t}^{\alpha }u\in L^{q}\left(\left(0,T\right);H^{-1}\left(\Omega \right)\right), \end{array}$$

and

$$\begin{array}{c} f\left(u^{m}\right)⇀\chi \;\;\;\;\mathrm{in}\;\mathrm{the}\;\mathrm{sense}\;\mathrm{of}\;\mathrm{weak}\;\mathrm{topology}\;\mathrm{of}\;L^{q}\left(\left(0,T\right);L^{q}\left(\Omega \right)\right), \end{array}$$

i.e.,

$$\begin{array}{c} \int_{0}^{T}\left(f\left(u^{m}\right),v\right)\to \int_{0}^{T}\left(\chi ,v\right)\;\;\;\;\mathrm{for}\;\mathrm{all}\;v\in L^{p}\left(\left(0,T\right);L^{p}\left(\Omega \right)\right),\;\frac{1}{p}+\frac{1}{q}=1. \end{array}$$

We need to identify that χ=f(u). By Corollary 1.12 in [30], we know that $u^{m}$ converges pointwise to *u* almost everywhere in Ω; therefore, by the continuity of *f*, we find that $f\left(u^{m}\right)\to f\left(u\right)$ almost everywhere. Using Lemma 8.3 in [30], we obtain $f\left(u^{m}\right)⇀f\left(u\right)$ in $L^{q}\left(\left(0,T\right);L^{q}\left(\Omega \right)\right)$. By Proposition 3.3 in [31], the space $L^{q}\left(\left(0,T\right);L^{q}\left(\Omega \right)\right)$ with weak topology is Hausdorff, then the limit is unique, which means that χ=f(u). Next, we need to prove that $g\left(t,u_{t}^{m}\right)\to g\left(t,u_{t}\right)$.

By a similar procedure as in Theorem 3.1 in [24], we can verify that $g\left(t,u_{t}^{m}\right)\to g\left(t,u_{t}\right)$ in $L^{2}\left(\left(0,T\right);L^{2}\left(\Omega \right)\right)$. Hence, *u* is a weak solution of (7) and (8).

Step 5. Uniqueness of solution. Let u(t;ϕ),v(t;ϕ) be two weak solutions of problem (7) and (8), with u(t)=v(t)=ϕ(t), t∈[−h,0]. Set w(t)=u(t)−v(t), t≥0, then w(t)=0, for all t∈[−h,0]. For w(t), we have

$$\begin{array}{c} D_{t}^{\alpha }w-\nu \Delta w=f\left(u\right)-f\left(v\right)+g\left(t,u_{t}\right)-g\left(t,v_{t}\right). \end{array}$$

Taking the inner product of the above equation with w(t), we obtain

$$\begin{array}{cc} D_{t}^{\alpha }{\left|w\right|}^{2}+2\nu {∥w∥}^{2} & =\left(f\left(u\right)-f\left(v\right),w\right)+\left(g\left(t,u_{t}\right)-g\left(t,v_{t}\right),w\right)\\ \; & \leq 2\alpha_{3}{\left|w\right|}^{2}+2L_{g}\underset{0\leq s\leq t}{\sup}{\left|w\left(s\right)\right|}^{2}\\ \; & \leq \left(2\alpha_{3}+2L_{g}\right)\underset{0\leq s\leq t}{\sup}{\left|w\left(s\right)\right|}^{2},\;\;\mathrm{for}\;\mathrm{all}\;t\in \left[0,T\right]. \end{array}$$

The above inequality holds true for any t∈[0,T], and then we have

$$\begin{array}{c} \omega \left(t\right)\leq \frac{\left(2\alpha_{3}+2L_{g}\right)}{\Gamma (\alpha )}\int_{0}^{t}{\left(t-s\right)}^{\alpha -1}\underset{0\leq r\leq s}{\sup}{\left|w\left(r\right)\right|}^{2}ds:=\left(2\alpha_{3}+2L_{g}\right)g_{\alpha }*\omega \left(t\right), \end{array}$$

where $\underset{0\leq s\leq t}{\sup}{\left|w\left(s\right)\right|}^{2}=\omega \left(t\right)$. Next, we convolve the above inequality with $g_{n_{0}\alpha }$, and have

$$\begin{array}{c} \omega_{1}\left(t\right)\leq \left(2\alpha_{3}+2L_{g}\right)g_{\alpha }*\omega_{1}\left(t\right), \end{array}$$

where $\omega_{1}\left(t\right)=g_{n_{0}\alpha }*\omega \left(t\right)$. Note that $g_{n_{0}\alpha }=Ct^{n_{0}\alpha -1}$; if $n_{0}$ is large enough, then $\omega_{1}\left(t\right)$ is continuous on [0,T]. By iteration—group property as well as Stirling formula, i.e., $\Gamma \left(n\alpha +1\right)=\sqrt{2\pi n\alpha }{\left(\frac{n\alpha }{e}\right)}^{n\alpha }exp\left(\frac{\gamma }{12n\alpha }\right)$ with γ∈(0,1)—we find

$$\begin{array}{cc} \omega_{1}\left(t\right) & \leq {\left(2\alpha_{3}+2L_{g}\right)}^{n}g_{n\alpha }*\omega_{1}\left(t\right)\\ \; & \leq \frac{{\left(2\alpha_{3}+2L_{g}\right)}^{n}}{\Gamma (n\alpha +1)}\underset{0\leq t\leq T}{\sup}\omega_{1}\left(t\right)\int_{0}^{t}{\left(t-s\right)}^{n\alpha }ds\to 0,\;\mathrm{as}\;n\;\mathrm{large}\;\mathrm{enough}. \end{array}$$

Then, $\omega_{1}\left(t\right)=0$ on [0,T]. Convolving both sides with $g_{-n_{0}\alpha }$ on $\omega_{1}\left(t\right)=g_{n_{0}\alpha }*\omega \left(t\right)$, to find that $\underset{0\leq s\leq t}{\sup}{\left|w\left(s\right)\right|}^{2}=0$ on [0,T].

Therefore, w(t)=0 on [−h,T]. The proof is complete. □

**Remark** **1.** *A detailed procedure of Step 3 in the above proof can be found in Theorem 3.1 in [24], in which the Banach–Alaoglu–Bourbaki theorem and the Eberlin–Smulian theorem [31] are applied.*

**Theorem** **2.** *Assume that (g1)–(g3) hold true, then the weak solutions of problems (1)–(3) are continuous, with respect to the initial values, i.e.,*

$$\begin{array}{c} ∥u_{t}-v_{t}{∥}_{C_{H}}^{2}\leq C\left(t\right){∥\phi \left(t\right)-\varphi \left(t\right)∥}^{2}. \end{array}$$

**Proof.**  Let u(t;ϕ), v(t;φ) be two weak solutions of (1)–(3), with initial values, ϕ and φ, respectively. Set w(t)=u(t)−v(t), for t>0, and w(t)=ϕ(t)−φ(t) for t∈[−h,0]. Then, we have

$$\begin{array}{c} D_{t}^{\alpha }w-\nu \Delta w=f\left(u\right)-f\left(v\right)+g\left(t,u_{t}\right)-g\left(t,v_{t}\right). \end{array}$$

Taking the inner product of the above equation with w(t), we obtain

$$\begin{array}{cc} D_{t}^{\alpha }{\left|w\right|}^{2}+\nu {∥w∥}^{2} & =\left(f\left(u\right)-f\left(v\right),w\right)+\left(g\left(t,u_{t}\right)-g\left(t,v_{t}\right),w\right)\\ \; & \leq \left(2\alpha_{3}+2L_{g}\right){∥w_{s}∥}_{C_{H}}^{2}. \end{array}$$

By a similar method as we used in Step 2 of Theorem 1, we find that

$$\begin{array}{c} {∥w∥}_{C_{H}}^{2}\leq C\left(t\right){∥\phi -\varphi ∥}^{2}. \end{array}$$

The proof is complete. □

**Remark** **2.** *Compared to [24], the restriction of $\alpha \in (0,\frac{1}{2})$ is removed here.*

## 4. A Priori Estimations: Existence of Global Attracting Set

We establish some estimates of weak solutions to system (1)–(3) by using Lemma 3 in this section. Henceforth, we assume that

(13) $$\lambda_{1}\nu >L_{g}.$$

**Lemma** **4** (Existence of absorbing sets in $C_{H}$). *Suppose that (g1)–(g3) and (13) hold true, then there exists T>0, such that for all t≥T the weak solution of problem (1)–(3) satisfies*

$$\begin{array}{c} ∥u_{t}{∥}_{C_{H}}^{2}\leq \rho_{C_{H}}^{2},\;t\geq T, \end{array}$$

*where $\rho_{C_{H}}^{2}=\frac{2\lambda_{1}\nu \beta_{2}\left|\Omega \right|}{{\left(\lambda_{1}\nu \right)}^{2}-L_{g}^{2}}.$*

**Proof.**  Taking the inner product of (1) with *u*, we have

$$\begin{array}{cc} D_{t}^{\alpha }{\left|u\left(t\right)\right|}^{2}+\lambda_{1}{\nu |u\left(t\right)|}^{2}+2\alpha_{2}{∥u∥}_{p}^{p} & \leq 2\beta_{2}\left|\Omega \right|+\frac{L_{g}^{2}}{\lambda_{1}\nu }\underset{t-\tau (t)\leq s\leq t}{\sup}{\left|u\left(s\right)\right|}^{2}; \end{array}$$

in other words,

$$\begin{array}{cc} D_{t}^{\alpha }{\left|u\left(t\right)\right|}^{2}\; & \leq 2\beta_{2}\left|\Omega \right|-\lambda_{1}{\nu |u\left(t\right)|}^{2}+\frac{L_{g}^{2}}{\lambda_{1}\nu }\underset{t-\tau (t)\leq s\leq t}{\sup}{\left|u\left(s\right)\right|}^{2},\;\;t\in \left(0,T\right],\\ \; & {\left|u\left(t\right)\right|}^{2}={\left|\phi \left(t\right)\right|}^{2},\;\;t\in \left[-h,0\right]. \end{array}$$

Using Lemma 3, we obtain

$$\begin{array}{c} {\left|u\left(t\right)\right|}^{2}\leq \frac{2\lambda_{1}\nu \beta_{2}\left|\Omega \right|}{{\left(\lambda_{1}\nu \right)}^{2}-L_{g}^{2}}+ME_{\alpha }\left(\lambda^{*}t^{\alpha }\right), \end{array}$$

for all t≥τ(t)+1, where $M={∥\phi \left(t\right)∥}_{C_{H}}^{2}=\underset{-h\leq t\leq 0}{\sup}{\left|\phi \left(t\right)\right|}^{2}$, and the parameter $\lambda^{*}$ is defined by

$$\begin{array}{c} \lambda^{*}=\underset{t-\tau (t)\geq 1}{\sup}\left\{\lambda :\lambda -\left(-\lambda_{1}\nu \right)-\frac{L_{g}^{2}}{\lambda_{1}\nu }\frac{E_{\alpha }\left(\lambda {\left(t-\tau \left(t\right)\right)}^{\alpha }\right)}{E_{\alpha }\left(\lambda t^{\alpha }\right)}=0\right\}, \end{array}$$

which is strictly negative, i.e, there exists some positive constants $\epsilon_{0}$ satisfying $-\lambda_{1}\nu +\frac{L_{g}^{2}}{\lambda_{1}\nu }<-\epsilon_{0}$, such that $\lambda^{*}\in \left[-\lambda_{1}\nu +\frac{L_{g}^{2}}{\lambda_{1}\nu },-\epsilon_{0}\right]$, and the estimate in (9) holds for all *t*, such that t≥τ(t)+1.

On the other hand, if t<τ(t)+1, then, by the same argument as in Theorem 4.1 in [24], we have

$$\begin{array}{c} {\left|u\left(t\right)\right|}^{2}\leq CE_{\alpha }\left(-\lambda_{1}\nu t^{\alpha }\right),\;\;t\geq 0,\theta \in \left[-h,0\right]. \end{array}$$

From the Archimedes principle, we conclude that

$$\begin{array}{c} ∥u_{t}{∥}_{C_{H}}^{2}\leq \frac{2\lambda_{1}\nu \beta_{2}\left|\Omega \right|}{{\left(\lambda_{1}\nu \right)}^{2}-L_{g}^{2}}+ME_{\alpha }\left(\lambda^{*}t^{\alpha }\right)+CE_{\alpha }\left(-\lambda_{1}\nu t^{\alpha }\right),\;\;0\leq t<\tau \left(t\right)+1. \end{array}$$

As $\lambda^{*}<0$ and $-\lambda_{1}\nu <0$, by the property of the Mittag-Leffler function [2], we obtain

$$\begin{array}{c} ∥u_{t}{∥}_{C_{H}}^{2}\leq \frac{2\lambda_{1}\nu \beta_{2}\left|\Omega \right|}{{\left(\lambda_{1}\nu \right)}^{2}-L_{g}^{2}}+C\frac{C_{\alpha }}{t^{\alpha }},\;\mathrm{as}\;t\to +\infty , \end{array}$$

where $C_{\alpha }>0$ is a constant independent of *t*. Hence, there exists T>0 large enough, such that ∀t≥T, the weak solution of problem (1)–(3), satisfies

$$\begin{array}{c} ∥u_{t}{∥}_{C_{H}}^{2}\leq \frac{2\lambda_{1}\nu \beta_{2}\left|\Omega \right|}{{\left(\lambda_{1}\nu \right)}^{2}-L_{g}^{2}}:=\rho_{C_{H}}^{2},\;t\geq T. \end{array}$$

Denote by $B_{C_{H}}=B\left(0,\rho_{C_{H}}\right)$ the absorbing set in phase space $C_{H}$. □

**Lemma** **5** (Existence of absorbing sets in $C_{V}$). *Suppose that (g1)–(g3) and (13) hold true; then, there exists T>0, such that for all t≥T, the weak solution of problems (1)–(3) satisfies*

$$\begin{array}{c} ∥u_{t}{∥}_{C_{V}}^{2}\leq \rho_{C_{V}}^{2},\;t\geq T+1, \end{array}$$

*where $\rho_{C_{V}}^{2}=\frac{\left(\Gamma \left(\alpha \right)+2\alpha_{3}\right)(2\beta_{2}\left|\Omega \right|+L_{g}^{2})+4\nu \lambda_{1}\alpha_{3}\beta_{2}\left|\Omega \right|L_{g}^{2}}{\Gamma \left(\alpha +1\right)(\nu +{\left(\lambda_{1}\nu \right)}^{2}-L_{g}^{2})}.$*

**Proof.**  Taking the inner product of (1) with −Δu, we find that

$$\begin{array}{cc} \frac{1}{2}D_{t}^{\alpha }{∥u∥}^{2}+\nu {\left|\Delta u\right|}^{2} & \leq \left(f\left(u\right),-\Delta u\right)+\left(g\left(t,u_{t}\right),-\Delta u\right)\\ \; & \leq \alpha_{3}{∥u∥}^{2}+\frac{\nu }{2}{\left|\Delta u\right|}^{2}+\frac{1}{2\nu }{\left|g\left(t,u_{t}\right)\right|}^{2}. \end{array}$$

Then,

$$\begin{array}{c} D_{t}^{\alpha }{∥u∥}^{2}+{\nu |\Delta u|}^{2}\leq 2\alpha_{3}{∥u∥}^{2}+\frac{L_{g}^{2}}{\nu }{∥u_{t}∥}_{C_{H}}^{2}, \end{array}$$

integrating above inequality over [s,t], to obtain

$$\begin{array}{c} {∥u\left(t\right)∥}^{2}-{∥u\left(s\right)∥}^{2}\leq \frac{2\alpha_{3}}{\Gamma (\alpha )}\int_{s}^{t}{\left(t-r\right)}^{\alpha -1}{∥u\left(r\right)∥}^{2}dr+\frac{L_{g}^{2}}{\nu \Gamma (\alpha )}\int_{s}^{t}{\left(t-r\right)}^{\alpha -1}{∥u_{r}∥}_{C_{H}}^{2}dr, \end{array}$$

and integrating with respect to *s* over interval [t−1,t] with t≥T+h+1, to yield

$$\begin{array}{cc} {∥u\left(t\right)∥}^{2} & \leq \int_{t-1}^{t}{∥u\left(s\right)∥}^{2}ds+\frac{2\alpha_{3}}{\Gamma (\alpha )}\int_{t-1}^{t}\int_{s}^{t}{\left(t-r\right)}^{\alpha -1}{∥u\left(r\right)∥}^{2}drds\\ \; & \;\;\;+\frac{L_{g}^{2}}{\nu \Gamma (\alpha )}\int_{t-1}^{t}\int_{s}^{t}{\left(t-r\right)}^{\alpha -1}{∥u_{r}∥}_{C_{H}}^{2}drds\\ \; & =\int_{t-1}^{t}{∥u\left(s\right)∥}^{2}ds+\frac{2\alpha_{3}}{\Gamma (\alpha )}\int_{t-1}^{t}\int_{t-1}^{r}{\left(t-r\right)}^{\alpha -1}{∥u\left(r\right)∥}^{2}dsdr\\ \; & \;\;\;+\frac{L_{g}^{2}}{\nu \Gamma (\alpha )}\int_{t-1}^{t}\int_{t-1}^{r}{\left(t-r\right)}^{\alpha -1}{∥u_{r}∥}_{C_{H}}^{2}dsdr\\ \; & =\int_{t-1}^{t}{∥u\left(s\right)∥}^{2}ds+\frac{2\alpha_{3}}{\Gamma (\alpha )}\int_{t-1}^{t}\left(r-t+1\right){\left(t-r\right)}^{\alpha -1}{∥u\left(r\right)∥}^{2}dr\\ \; & \;\;\;+\frac{L_{g}^{2}}{\nu \Gamma (\alpha )}\int_{t-1}^{t}\left(r-t+1\right){\left(t-r\right)}^{\alpha -1}{∥u_{r}∥}_{C_{H}}^{2}dsdr\\ \; & \leq \int_{t-1}^{t}{∥u\left(s\right)∥}^{2}ds+\frac{2\alpha_{3}}{\Gamma (\alpha )}\int_{t-1}^{t}{\left(t-r\right)}^{\alpha -1}{∥u\left(r\right)∥}^{2}dr\\ \; & \;\;\;+\frac{L_{g}^{2}}{\nu \Gamma (\alpha )}\int_{t-1}^{t}{\left(t-r\right)}^{\alpha -1}{∥u_{r}∥}_{C_{H}}^{2}dsdr\\ \; & \leq \left(1+\frac{2\alpha_{3}}{\Gamma (\alpha )}\right)\int_{t-1}^{t}{\left(t-r\right)}^{\alpha -1}{∥u\left(r\right)∥}^{2}dr+\frac{4\nu \lambda_{1}\alpha_{3}\beta_{2}\left|\Omega \right|L_{g}^{2}}{\left({\left(\lambda_{1}\nu \right)}^{2}-L_{g}^{2}\right)\Gamma \left(\alpha \right)}\int_{t-1}^{t}{\left(t-r\right)}^{\alpha -1}dr\\ \; & \leq \left(1+\frac{2\alpha_{3}}{\Gamma (\alpha )}\right)\left(\frac{2\beta_{2}\left|\Omega \right|}{\alpha \nu }+\frac{L_{g}^{2}}{\alpha \nu }\right)+\frac{4\nu \lambda_{1}\alpha_{3}\beta_{2}\left|\Omega \right|L_{g}^{2}}{\left({\left(\lambda_{1}\nu \right)}^{2}-L_{g}^{2}\right)\Gamma \left(\alpha +1\right)}\\ \; & =\frac{\left(\Gamma \left(\alpha \right)+2\alpha_{3}\right)(2\beta_{2}\left|\Omega \right|+L_{g}^{2})+4\nu \lambda_{1}\alpha_{3}\beta_{2}\left|\Omega \right|L_{g}^{2}}{\Gamma \left(\alpha +1\right)(\nu +{\left(\lambda_{1}\nu \right)}^{2}-L_{g}^{2})}. \end{array}$$

Therefore, ∀t≥T+2h+1, and we deduce that

$$\begin{array}{c} ∥u_{t}{∥}_{C_{V}}^{2}\leq \frac{\left(\Gamma \left(\alpha \right)+2\alpha_{3}\right)(2\beta_{2}\left|\Omega \right|+L_{g}^{2})+4\nu \lambda_{1}\alpha_{3}\beta_{2}\left|\Omega \right|L_{g}^{2}}{\Gamma \left(\alpha +1\right)(\nu +{\left(\lambda_{1}\nu \right)}^{2}-L_{g}^{2})}:=\rho_{C_{V}}^{2}. \end{array}$$

□

**Theorem** **3.** *Assume that (4)–(6), (g1)–(g3) and (13) hold true; then, the system (1)–(3) possesses a global attracting set in $C_{H}$.*

**Proof.**  Let $\left\{u^{n}\right\}$ with $u^{n}=u\left(t_{n};0,\phi^{n}\right)$ be a sequence of weak solutions to problem (1), defined on [−h,∞), with initial value $\phi^{n}\in B_{C_{H}}$, and $t_{n}\to \infty$ as n→∞.

On the one hand, by Lemmas 4 and 5, we know that the system (1)–(3) has a global absorbing set in $C_{H}$ and $C_{V}$, respectively. If we could show that $\left\{u_{t_{n}}^{n}\right\}$ is compact in $C_{H}$, then by attractor theory [30], we conclude that the system (1)–(3) possesses a global attracting set in $C_{H}$.

We use the Arzelà–Ascoli Theorem to prove this, i.e., we need to verify the following two conditions:

(i) for any sequence $\left\{u^{n}\right\}$ of weak solutions of (1) with initial value $\phi^{n}\in B_{C_{H}}$, we can find a sub-sequence denoted as $\left\{u^{n}\right\}$, such that $\left\{u_{t_{n}}^{n}\right\}$ is pre-compact in *H* for all θ∈[−h,0];
(ii) the sequence $\left\{u_{t_{n}}^{n}\right\}$ is equi-continuous with respect to θ∈[−h,0].

By Lemmas 4 and 5 and the compact embedding V↪H, we conclude that there exists N∈N, such that for all n≥N, $t_{N}\geq T+2h+1$, it holds that $\left\{u_{t_{n}}^{n}\right\}$ is compact in *H* for any θ∈[−h,0].

Let $\theta_{1},\theta_{2}\in \left[-h,0\right]$, without loss of generalization, we assume that $\theta_{1}\leq \theta_{2}$; note that

$$\begin{array}{cc} |u\left(t+\theta_{2}\right)-u\left(t+\theta_{1}\right)| & =|\frac{1}{\Gamma (\alpha )}\int_{t+\theta_{1}}^{t+\theta_{2}}{\left(t+\theta_{2}-s\right)}^{\alpha -1}D_{s}^{\alpha }u\left(s\right)ds|\\ \; & \leq \frac{1}{\Gamma (\alpha )}\int_{t+\theta_{1}}^{t+\theta_{2}}{\left(t+\theta_{2}-s\right)}^{\alpha -1}\left|\nu \Delta u\left(s\right)+f\left(u\right)+g\left(s,u_{s}\right)\right|ds\\ \; & \leq \frac{1}{\Gamma (\alpha )}\int_{t+\theta_{1}}^{t+\theta_{2}}{\left(t+\theta_{2}-s\right)}^{\alpha -1}(|\nu \Delta u\left(s\right)|+|f\left(u\right)|+|g\left(s,u_{s}\right)\left|\right)ds\\ \; & \leq C|\theta_{2}-\theta_{1}{|}^{\alpha }. \end{array}$$

Then, conclusion (ii) is proved immediately. Hence, $\left\{u_{t_{n}}^{n}\right\}$ is compact in $C_{H}$, using the theory of [30], and we conclude that system (1) has a global attracting set.

**Remark** **3.** *The global attracting set is obtained, which is different from its integer counterparts, such as [32], in which the existence of a pullback attractor is obtained.*

□

## 5. Conclusions

In this work, we researched the fractional reaction–diffusion equation with bounded delay, in the sense of weak topology. The existence and uniqueness of a weak solution and global attracting sets were proved. Compared to the classic evolution equation with delay (bounded or unbounded), the study of the limit behavior of the time-fractional evolution equation with unbounded delay is much more complicated. As the derivative is not integer, methods that are used to analyze classic evolution equation do not work anymore. To detect the limit behavior of the time-fractional evolution equation with unbounded delay, we need to explore new techniques. We studied the dynamics of fractional Navier–Stokes equations in our former work [24]; however, we could not prove the existence of a global attracting set even in the non-delay case, let alone the delay case. It is complicated to prove the existence of a weak solution in the delay case; therefore, in the future, we will also work on fractional delayed Navier–Stokes equations.

## Data Availability

The data presented in this study are available on request from the corresponding author.
